# TIR Domain-Containing Adaptor-Inducing Interferon-β (TRIF) Participates in Antiviral Immune Responses and Hepatic Lipogenesis of Large Yellow Croaker (*Larimichthys Crocea*)

**DOI:** 10.3389/fimmu.2019.02506

**Published:** 2019-10-30

**Authors:** Si Zhu, Xiaojun Xiang, Xiang Xu, Shengnan Gao, Kangsen Mai, Qinghui Ai

**Affiliations:** ^1^Key Laboratory of Aquaculture Nutrition and Feed (Ministry of Agriculture), Key Laboratory of Mariculture (Ministry of Education), College of Fisheries, Ocean University of China, Qingdao, China; ^2^Laboratory for Marine Fisheries and Aquaculture, Qingdao National Laboratory for Marine Science and Technology, Qingdao, China

**Keywords:** *Larimichthys crocea*, TRIF, poly (I:C), antiviral immune responses, hepatic lipogenesis

## Abstract

TIR domain-containing adaptor-inducing interferon-β (TRIF), a cytosolic adaptor protein, plays a key role in the mammalian toll-like receptor-mediated signaling pathway. However, the role of TRIF in large yellow croaker (LcTRIF) remains poorly understood. The main objective of this study was to explore the role of LcTRIF in triggering antiviral immune responses and the potential function of LcTRIF in regulating lipid metabolism. In the present study, the full-length coding sequence of *TRIF* from large yellow croaker was cloned and characterized. *In vivo*, upon poly (I:C) stimulation, the transcriptional levels of *LcTRIF* were rapidly elevated in immune-related tissues at the early stage of injection. *In vitro*, the MRNA expression of *LcTRIF* was significantly but not dramatically upregulated in macrophages treated with poly (I:C). Activation of *LcTRIF* by poly (I:C) significantly increased the transcription of genes involved in inflammatory responses, and this induction was blocked by knockdown of *LcTRIF*. Moreover, the ability of LcTRIF to induce inflammatory responses may partially be attributed to the promotion of mRNA expression of IFNh and NF-κB pathway genes. In addition, activation of the LcTRIF-mediated pathway inhibited the increase in hepatic stearoyl-coenzyme A (CoA) desaturase 1 induced by palmitic acid and subsequently alleviated lipid accumulation in hepatocytes. These results revealed the crucial role of LcTRIF in triggering antiviral immune responses and the unconventional metabolic function of LcTRIF in regulating hepatic lipogenesis in large yellow croaker.

## Introduction

Toll-like receptors (TLRs), one of the most extensively studied pattern-recognition receptors (PRRs), play a crucial role in both the innate immune system and the adaptive immune system by recognizing conserved components of pathogens referred to as pathogen associated molecular patterns (PAMPs) (1, 2). Upon stimulation with PAMPs, TLRs selectively recruit distinct TIR domain-containing adaptor molecules and induce specific immune responses to kill invading pathogens (3, 4). To date, six adaptors have been identified in mammals, including myeloid differentiation primary response gene 88 (MyD88), TIR domain-containing adaptor-inducing interferon-β (TRIF), TIR domain-containing adaptor protein (TIRAP), TRIF-related adaptor molecule (TRAM), sterile α and armadillo motif-containing protein 1 (SARM1), and B cell adaptor for phosphoinositide 3-kinase (BCAP).

TRIF, also known as TIR domain-containing adapter molecule 1 (TICAM-1), is unique to TLR3-and TLR4-mediated signaling pathways, which activate interferon (IFN) regulatory factors 3/7 (IRF3/7) and nuclear factor-kappa B (NF-κB) and induce the production of type I IFN and inflammatory cytokines, leading to direct killing of invading pathogens (5–7). An increasing number of studies have recognized that apart from its typical role in triggering antiviral immune responses, the TRIF-dependent TLR pathway also plays a distinct role in lipid metabolism that is associated with various metabolic diseases in metabolic cells (8–10). In mammals, TRIF possesses proline-rich domains on the N- and C-terminal regions and a highly conserved TIR domain. Each domain plays a distinct role in signal transduction. The N-terminal domain contains a binding site for tumor necrosis factor receptor-associated factor (TRAF) proteins, which is crucial for IRF3 activation (6, 11). The C-terminal domain harbors receptor-interacting protein 1 (RIP1) interaction motif (RHIM), which is crucial for NF-κB activation and apoptosis (12–14). The TIR domain of TRIF is responsible for interacting with the TIR domain of TLR3 as well as the TLR4–bridging adaptor TRAM (5, 7, 15). However, in teleosts, TRIF lacks both the N-terminal and the C-terminal proline-rich domains and the proline in box2 of the TIR domain, which suggests that fish TRIF activates type I IFN and NF-κB through different ways with its mammalian TRIF orthologs (16–19). These findings revealed a real difference in TRIF-mediated antiviral immune responses between teleosts and mammals. To date, TRIF homologs have been identified in several fish species, and their roles in antiviral immune responses have been investigated (16, 18–20). However, the unconventional function of the TRIF-mediated pathway in regulating lipid metabolism has not been reported in fish.

Large yellow croaker (*Larimichthys crocea*) is an economically important mariculture fish species in China. The production of this fish species was 177640 t in China in 2017, which has continued to hold first place in the mariculture output for several years (21). A high-fat diet (HFD) has been commonly used in the culture of large yellow croaker owing to its protein sparing effect. However, long-term intake of HFD often leads to abnormal lipid accumulation accompanied by low-grade, chronic inflammation (22, 23), which is similar to the hepatic steatosis observed in mammals, and results in a high mortality rate and diminishes the health benefits of fish for human consumption. In the present study, our main objective was to explore the role of LcTRIF in triggering antiviral immune responses and the potential function of LcTRIF in regulating lipid metabolism. Understanding the functions of TRIF in large yellow croaker may contribute to the development of management strategies for defense against virus infections, and alleviate the abnormal lipid accumulation and inflammation induced by a HFD.

## Materials and Methods

### Fish, Challenge, and Sample Collection

Juveniles of large yellow croaker, *Larimichthys crocea* (50.6 g ± 7.8 g) obtained from Ningbo, China, were reared in a circulating seawater system at 24–28°C. After 1 week of acclimation, the fish were used for the induction experiments. Fish were injected intraperitoneally with 0.25 mL of 1 mg/mL polyinosinic: polycytidylic acid [poly (I:C), Sigma, USA] or 0.25 mL of phosphate buffered saline (PBS, pH 7.4, Gibco). Tissues including the gill, head kidney, liver and spleen were collected from four fish at different time points (0, 6, 12, 24, and 48 h), and frozen immediately in liquid nitrogen, and then stored at −80°C for further analysis. Husbandry and handling of fish in the present study were performed strictly according to the Management Rule of Laboratory Animals (Chinese Order No. 676 of the State Council, revised 1 March 2017) and the Guidelines for the Use of Fishes in Research (24).

### Cells Culture and Reagents

Macrophages were isolated from the head kidney of large yellow croaker by using Percoll (Pharmacia, USA) density gradients according to our previous report (25). Macrophages were seeded into 6-well plates at a density of 2 × 10^6^ cells/mL and were cultured in 95% DMEM/F12 medium (BI, Israel) supplemented with 5% FBS (BI, Israel) and antibiotics (Gibco, USA) in a 5% CO_2_ atmosphere at 26°C. After 24 h of cultivation, macrophages were washed with PBS, transfected with or without small interfering RNAs (siRNAs) for 48 h, and then incubated with 5 μg/mL poly (I:C) for 2 h on the basis of the inflammatory macrophage model previously established by ourselves (26).

Primary hepatocytes of large yellow croaker were isolated and cultivated based on our previous report (27). Hepatocytes were seeded into 6-well plates at a density of 2 × 10^6^ cells/mL and were maintained in DMEM/F12 medium containing 15% FBS and antibiotics in a 5% CO_2_ atmosphere at 28°C. After 2 d of cultivation, the cells were washed with PBS and incubated with FBS free medium prior to treatment with or without 800 μM palmitic acid (PA) conjugated with fatty acid free BSA (Equitech-Bio, USA) and 5 μg/mL poly (I:C) for 6 h (for gene expression) or 12 h (for lipid droplet visualization and TAG quantification).

HEK293T cells were cultured in 90% DMEM, high glucose medium (BI, Israel) supplemented with 10% FBS and antibiotics in a 5% CO_2_ atmosphere at 37°C.

### Cloning and Sequence Analysis of the *LcTRIF* Gene

Primers for the amplification of partial cDNA were designed according to the large yellow croaker genomic sequence and the predicted sequence of *LcTRIF* (Supplementary Table 1). RACE primers were designed based on the partial *LcTRIF* cDNA sequence to clone the 3' and 5'-end sequences through nest-PCR using the SMARTer^TM^ RACE cDNA Amplification Kit (Clontech, USA) (Supplementary Table 1). The full length of *LcTRIF* was assembled by overlapping the 5'-end sequence, partial cDNA sequence and 3'-end sequence and then was confirmed by sequencing the PCR product amplified by primers *TRIF*-FL-F and *TRIF*-FL-R (Supplementary Table 1). The nucleotide and deduced amino acid sequences of *LcTRIF* were analyzed by DNAMAN. Sequence similarity analysis was conducted using the BLAST program (https://blast.ncbi.nlm.nih.gov/Blast.cgi). The prediction of the protein domain was performed by SMART (http://smart.embl-heidelberg.de/). Multiple alignments of amino acid sequences from different species were analyzed using DNAMAN, and the phylogenetic tree was constructed by the neighbor-joining method in MEGA.

### Quantification of Gene Expression

Total RNA extraction, cDNA synthesis and quantitative real-time PCR (qRT-PCR) were carried out according to our previous report (28). The primer sequences for *TRIF*, stearoyl–coenzyme A (CoA) desaturase 1 (*SCD1*), fatty acid synthase (*FAS*), sterol-regulatory element binding protein 1 (*SREBP1*), diacylgycerol acyltransferase 2 (*DGAT2*), acetyl-CoA carboxylase 1 (*ACC1*), *IFNh, IRF3*, interleukin 1β (*IL-1*β), tumor necrosis factor α (*TNF*α), and β*-actin* are listed in Supplementary Table 2. β*-actin, GAPDH, 18S rRNA*, and *ubiquitin* were selected to test their suitability for the normalization of gene expression levels in various tissues of large yellow croaker. NormFinder algorithms and geNorm were further used to verify the stability and suitability of these genes (29, 30). No significant differences in β*-actin* expression were detected among all treatments, suggesting that β*-actin* could be used as a reference gene in the present study. The mRNA expression of each gene was normalized to that of β*-actin* using the 2^−ΔΔt^ method (31).

### Plasmid Construction

PCR was performed with primers listed in Supplementary Table 3 for various plasmid construction. In detail, for the expression plasmid, the coding sequence (CDS) of *LcTRIF* was cloned from large yellow croaker cDNA, the 5′ and 3′ ends homologous arms of the pCS2+ vector (5′ ends: 5′-CGATTCGAATTCAAGGCCTCTCGAG- 3′; 3′ ends: 5′-CTCACTATAGTTCTAGAGGCTCGAG- 3′) designed by TSINGKE Biotech Co., Ltd. (Qingdao, China) were added to the *LcTRIF* CDS by PCR. A linearized pCS2+ vector was obtained by digestion with QuickCut™ XhoI (Takara, Japan). Then, the CDS of *LcTRIF* was inserted into the XhoI site of the pCS2+ vector by homologous recombination using the ClonExpress® II One Step Cloning Kit (Vazyme Biotech Co., Ltd, Nanjing, China) to construct the pCS2-TRIF expression plasmid. For reporter plasmids, the *IFNh* promoter (1,324 bp genomic fragment, GenBank Accession No: KU144879.1), *NF-*κ*B* response promoter (2,208 bp genomic fragment, GenBank Accession No: NM_001303377.1) and *SCD1* promoter (2,911 bp genomic fragment, GenBank Accession No: KP202156.1) were cloned, the 5' and 3' ends homologous arms of the pGL3 Basic vector (5′ ends: 5′-TCTTACGCGTGCTAGCCCGGGC- 3′; 3′ ends: 5′-GCCAAGCTTACTTAGATCGCAGATC- 3') were added to the promoter sequences by PCR. Then, the promoters of *IFNh, NF-*κ*B* and *SCD1* were each subcloned into the XhoI site of the linearized pGL3-basic vector to construct the pGL3-IFNh, pGL3-NF-κB, and pGL3-SCD1 plasmids, respectively. For the subcellular localization studies, truncated forms of TRIF including the N terminus (1–350 aa), the TIR domain (351–471 aa), the C terminus (472–601 aa), ΔC (1–471 aa), and ΔN (351–601 aa) were subcloned into pcDNA3.1-EGFP vector. Plasmids used for transfection in the present study were purified using the EasyPure HiPure Plasmid MiniPrep Kit (TransGen Biotech Co., Ltd, Beijing, China) and were tested by sequencing at Sangon Biotech Co., Ltd. (Shanghai, China).

### RNA Interference

To knockdown the expression of *LcTRIF*, an RNA interference assay was conducted by transfecting siRNAs targeting the *LcTRIF* mRNA. Four siRNA sequences (Supplementary Table 4) were designed and synthesized by GenePharma (Shanghai, China). Macrophages were mock-transfected or transfected with siRNAs using Lipofectamine® RNAiMAX Transfection Reagent (Invitrogen, USA) for 48 h according to the manufacturer's instructions, and the knockdown efficiencies of the siRNAs were examined by qRT-PCR (Supplementary Figure 1).

### Dual-Luciferase Reporter Assays

HEK293T cells (5 × 10^5^ cells/mL) were cultured in 24-well plates overnight and transiently cotransfected with the IFNh promoter luciferase reporter plasmid, NF-κB response promoter luciferase reporter plasmid or SCD1 promoter luciferase reporter plasmid, together with the LcTRIF expression plasmid using Lipofectamine 3000 Reagent (Invitrogen, USA). After 24 h of transfection, the cells were stimulated with or without poly (I:C). After 24 h incubation, cells were lysed and analyzed for luciferase activity by the Dual-Luciferase Reporter Assay System (TransGen Biotech Co., Ltd, Beijing, China).

### BODIPY 493/503 Staining and Triglyceride (TAG) Content Quantification

Hepatocytes isolated from large yellow croaker were fixed with 4% paraformaldehyde (Solarbo, China) and incubated with BODIPY 493/503 (Invitrogen, USA) in the dark for 15 min. Then, lipid droplets were visualized by fluorescence microscopy (Leica, Germany). TAG content in the hepatocytes were quantified by a TAG Assay Kit according to the manufacturer's instructions (Applygen Technologies Inc., Beijing, China).

### Confocal Laser Microscopy Imaging

For the localization of LcTRIF, HEK293T cells (1.0 × 10^5^ cells/well) were seeded into coverslips in 24-well plates. The following day, cells were transiently transfected with pcDNA3.1-EGFP-TRIF vector or various truncation vectors using Lipofectamine 3000 Reagent (Invitrogen, USA). After 24 h, the cells were fixed with 4% paraformaldehyde and stained with DAPI (Solarbio, China) in the dark for 10 min. Thereafter, cells on coverslips were mounted onto a slide glass using PBS containing 2.3% 1,4-diazabicyclo [2.2.2]octane (DABCO) and 50% glycerol. Cells were visualized at × 60 magnification under an NIKON A1+ Confocal Laser Microscope (NIKON, Japan).

### Statistical Analysis

SPSS 20.0 was used to perform the statistical analysis. Values are presented as mean ± SD of three independent experiments with 4 technical replicates for each experiment. Data were processed using one-way ANOVA, followed by Tukey's Test. A value of *P* < 0.05 was considered statistically significant.

## Results

### Identification of the *TRIF* Gene in Large Yellow Croaker

**Figure 1:**
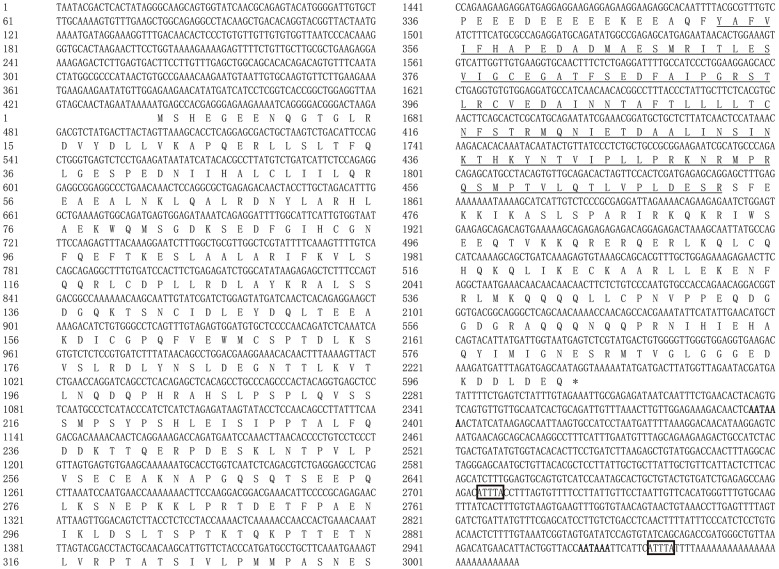
Nucleotide and deduced amino acid sequences of TRIF in large yellow croaker. The TIR domain is underlined. The two poly(A) signals (AATAAA) are highlighted in bold, and the two instability signals (ATTTA) are marked by boxes.

**Figure 2:**
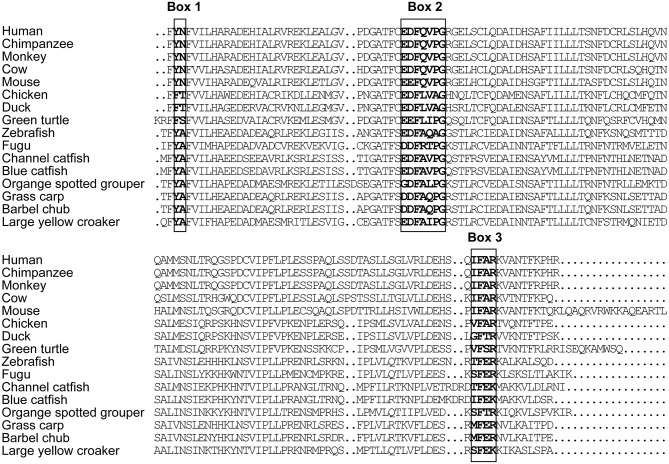
Alignment of TRIF TIR domain sequences from human (accession number NP_891549.1), chimpanzee (accession number NP_001123604.1), monkey (accession number AAS20428.1), cow (accession number NP_001025472.1), mouse (accession number AAH33406.1), chicken (accession number NP_001074975.1), duck (accession number NP_001297720.1), green turtle (accession number XP_007072279.1), zebrafish (accession number ABP04048.1), fugu (accession number NP_001106665.1), channel catfish (accession number NP_001187154.1), blue catfish (accession number ABH10662.1), orange spotted grouper (accession number AEX01719.1), grass carp (accession number AGW25589.1), and barbell chub (accession number AMP81962.1).

**Figure 3:**
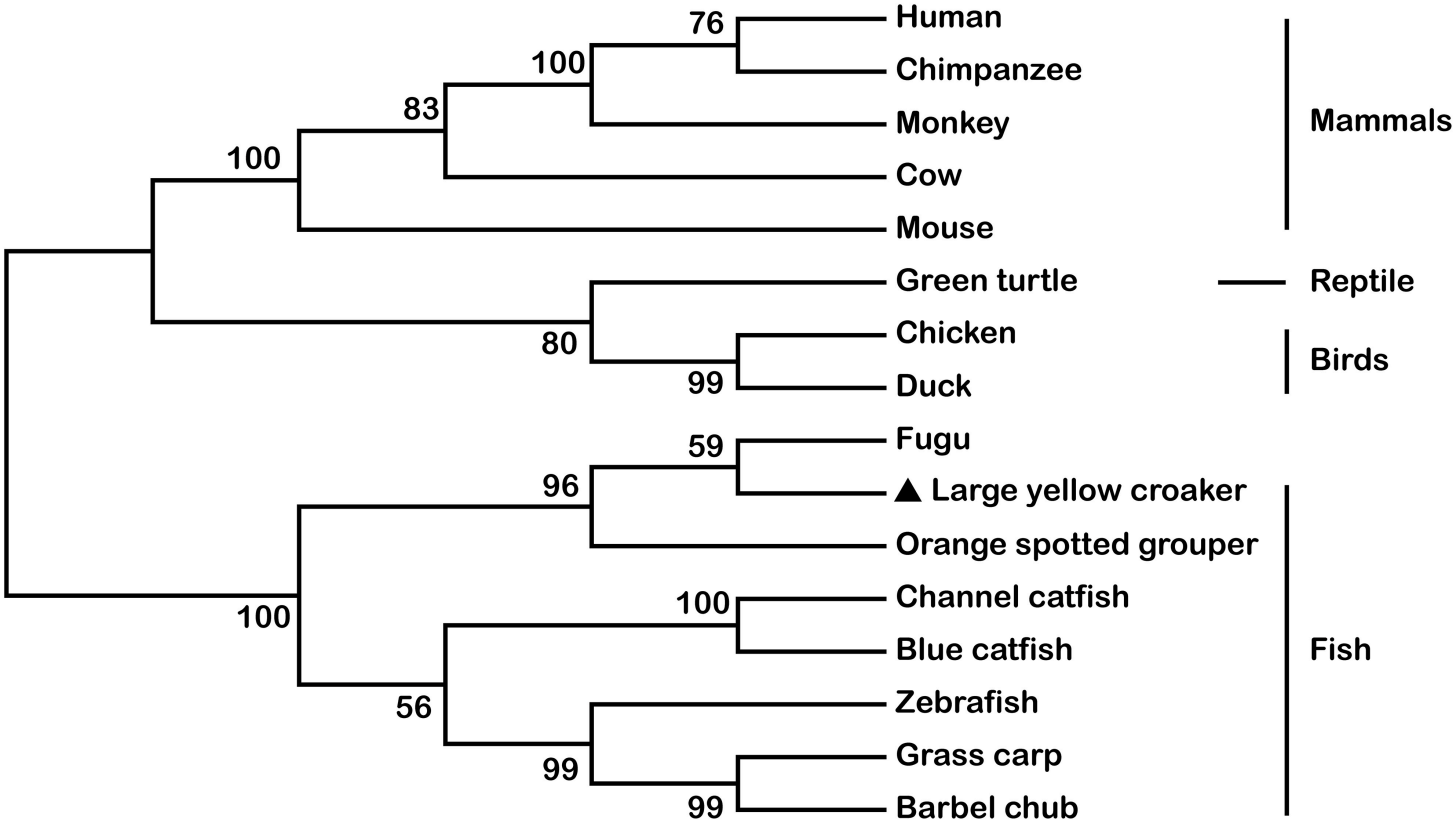
Phylogenetic tree based on the sequences of TRIF TIR domains from mammals, reptiles, birds, and other fish species. The accession numbers for the TIR domains of TRIF are shown in Figure 2.

The full-length cDNA of *LcTRIF* (GenBank Accession No: MH820380.1) was 3,012 bp and contained a 5′ untranslated terminal region (UTR) of 438 bp, a 3′ UTR of 768 bp, and an open reading frame (ORF) of 1,806 bp encoding a polypeptide of 601 amino acids (aa), which exhibited the typical characteristics of a TIR domain (351–471 aa) close to its C terminus. Two polyadenylation signals (AATAAA) were located 16 and 584 bp upstream of the poly(A) tail, respectively, and two instability signals (ATTTA) were found in the 3′ UTR (Figure 1). Multiple sequence alignment of the TIR domain of large yellow croaker TRIF with that of other species found that LcTRIF had three highly conserved regions: box 1 (YN), box 2 (EDFQVPG), and box 3 (IFAR) (Figure 2). The phylogenetic tree constructed based on the TIR domain of TRIF from different species showed that fish TRIF members formed an independent cluster, and LcTRIF was genetically closest to fugu TRIF (Figure 3).

### Modulation of *LcTRIF* mRNA Expression in Response to Poly (I:C) Stimulation *in vivo*

**Figure 4:**
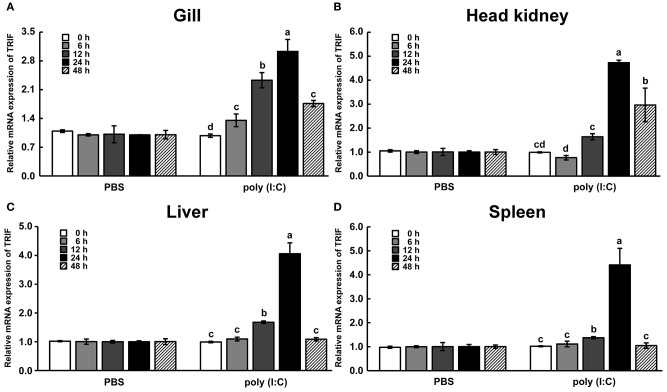
LcTRIF mRNA expression in immune-related tissues: gill **(A)**, head kidney **(B)**, liver **(C)**, and spleen **(D)** at different time points post poly (I:C) stimulation in large yellow croaker. Values (mean ± SD of three independent experiments with 4 technical replicates each) in bars that have the same superscript letter are not significantly different (*P* > 0.05, Tukey's test).

To investigate the temporal expression of *LcTRIF* in response to poly (I:C) (a dsRNA analog) stimulation, the mRNA levels of *LcTRIF* in immune-related tissues (gill, head kidney, liver, and spleen) were detected at different time points post injection (0, 6, 12, 24, and 48 h). In the gill, the mRNA levels of *LcTRIF* were significantly elevated at 6 h and reached maximum levels at 24 h (*P* < 0.05). In the head kidney, liver, and spleen, *LcTRIF* mRNA expression was obviously upregulated by poly (I:C) stimulation at 12 h and reached maximum levels at 24 h (*P* < 0.05) (Figure 4). No significant differences in *LcTRIF* mRNA expression were found in those tissues at different time points after injection with PBS (*P* > 0.05).

### Activation of LcTRIF by Poly (I:C) Induced Inflammatory Response *in vitro*

**Figure 5:**
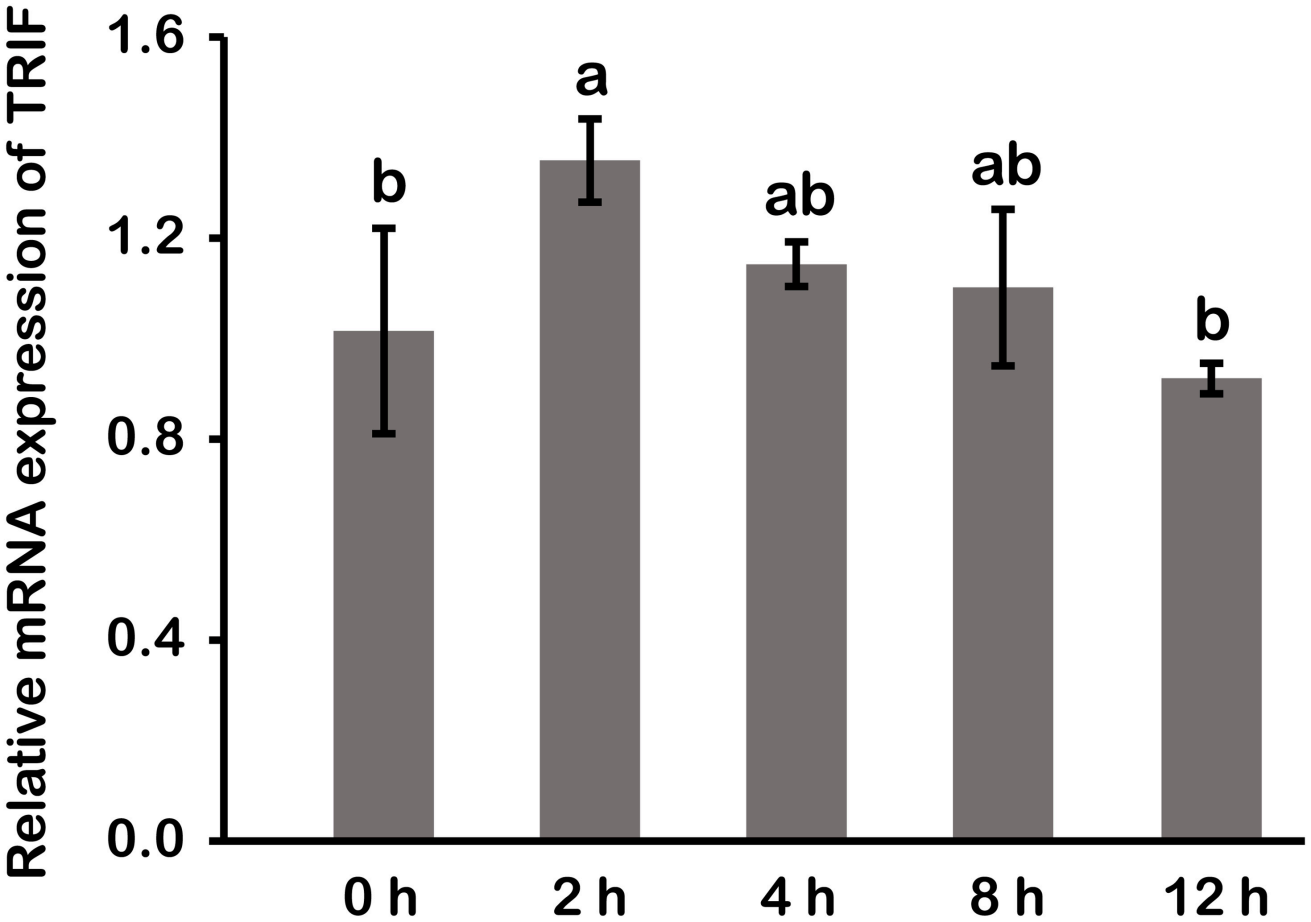
mRNA expression of LcTRIF in macrophages of large yellow croaker at different time points after poly (I:C) stimulation. Values (mean ± SD of three independent experiments with 4 technical replicates each) in bars that have the same superscript letter are not significantly different (*P* > 0.05, Tukey's test).

**Figure 6:**
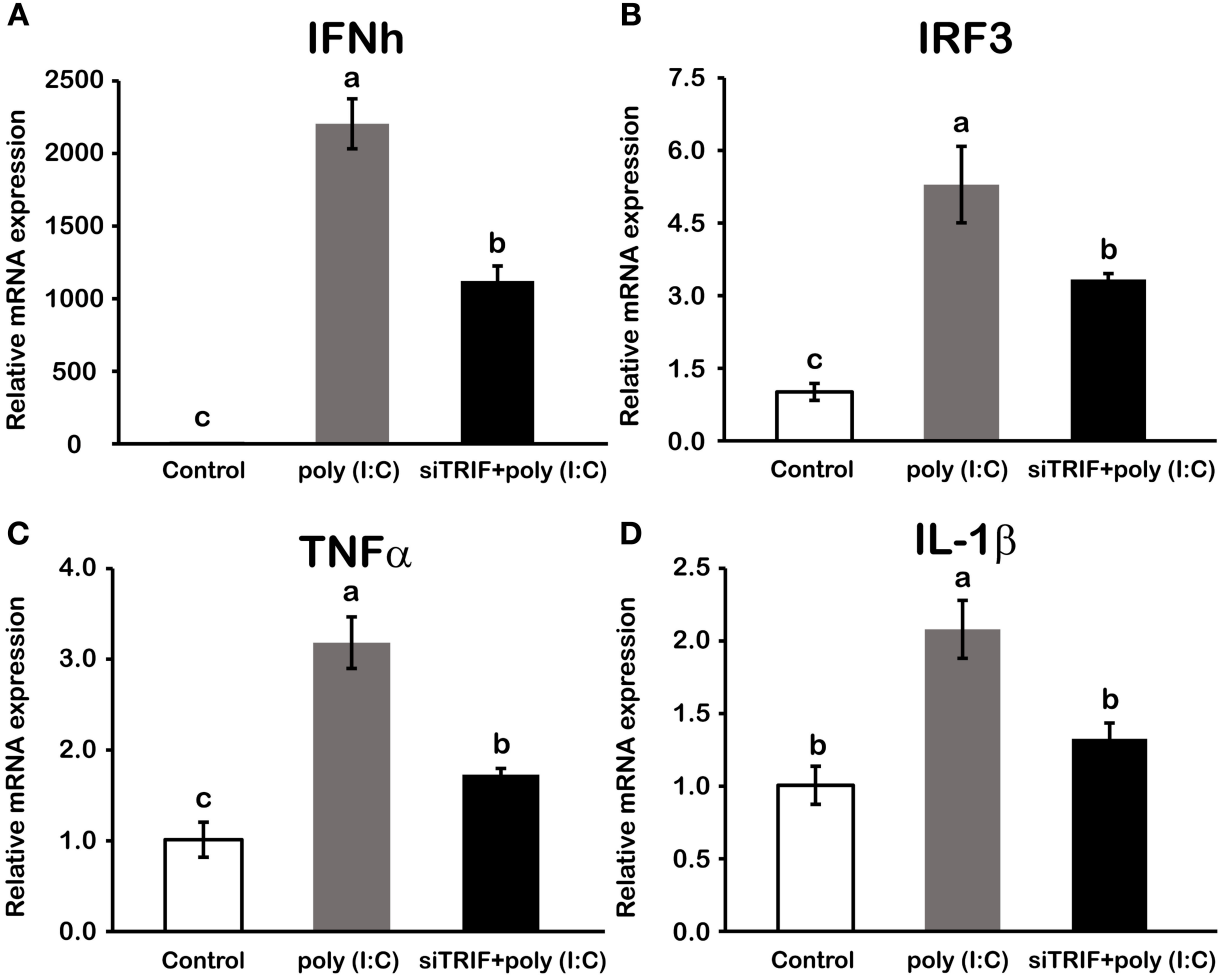
Activation of LcTRIF by poly (I:C) induced the mRNA expression of genes involved in the macrophage inflammatory response of large yellow croaker. Macrophages cultured in 6-well plates overnight were transfected with or without siTRIF and control siRNA. After 48 h, cells were stimulated with 5 μg/mL poly (I:C) for 2 h. The mRNA expression of IFNh **(A)**, IRF3 **(B)**, TNFα **(C)**, and IL-1β **(D)** were examined by qRT-PCR. Values (mean ± SD of three independent experiments with 4 technical replicates each) in bars that have the same superscript letter are not significantly different (*P* > 0.05, Tukey's test). IFNh, interferon h; IRF3, interferon regulatory factors 3; TNFα, tumor necrosis factor α; IL-1β, interleukin 1β.

In macrophages, mRNA expression of *LcTRIF* was rapidly increased in response to poly (I:C) stimulation at 2 h (*P* < 0.05), then gradually decreased over time, and returned to the control level at 12 h (Figure 5). In addition, activation of *LcTRIF* by poly (I:C) significantly elevated the transcription of genes involved in inflammatory responses, including *IFNh, IRF3, TNF*α, and *IL-1*β (*P* < 0.05), and the ability of poly (I:C) to induce the expression of these genes in macrophages was blocked by knockdown of *LcTRIF* with siRNA (*P* < 0.05) (Figure 6).

**Figure 7:**
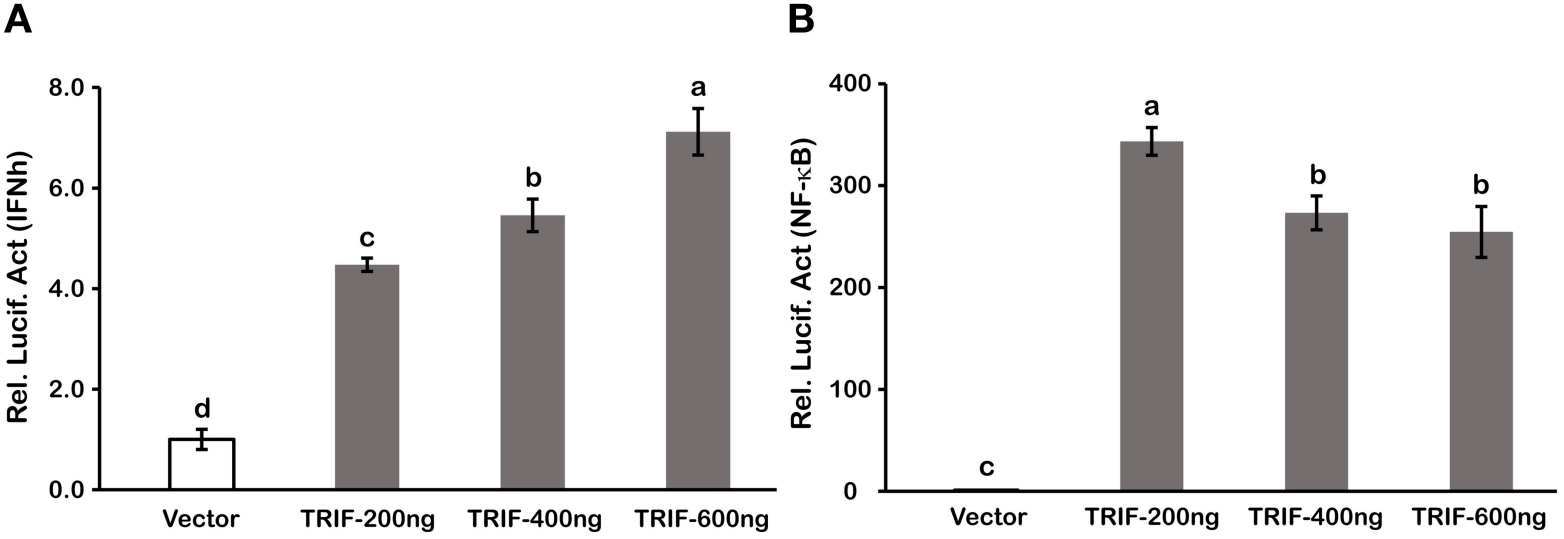
LcTRIF overexpression induced promoter activities of IFNh **(A)** and NF-κB **(B)**. HEK293T cells cultured in 24-well plates overnight were cotransfected with PCS2 empty vector or increasing amounts (200, 400, and 600 ng) of TRIF expression vector together with 200 ng of IFNh or NF-κB promoter luciferase reporter vector and 20 ng of pRL-TK. Twenty-four hours later, the luciferase activities were measured. Values (mean ± SD of three independent experiments with 4 technical replicates each) in bars that have the same superscript letter are not significantly different (*P* > 0.05, Tukey's test).

**Figure 8:**
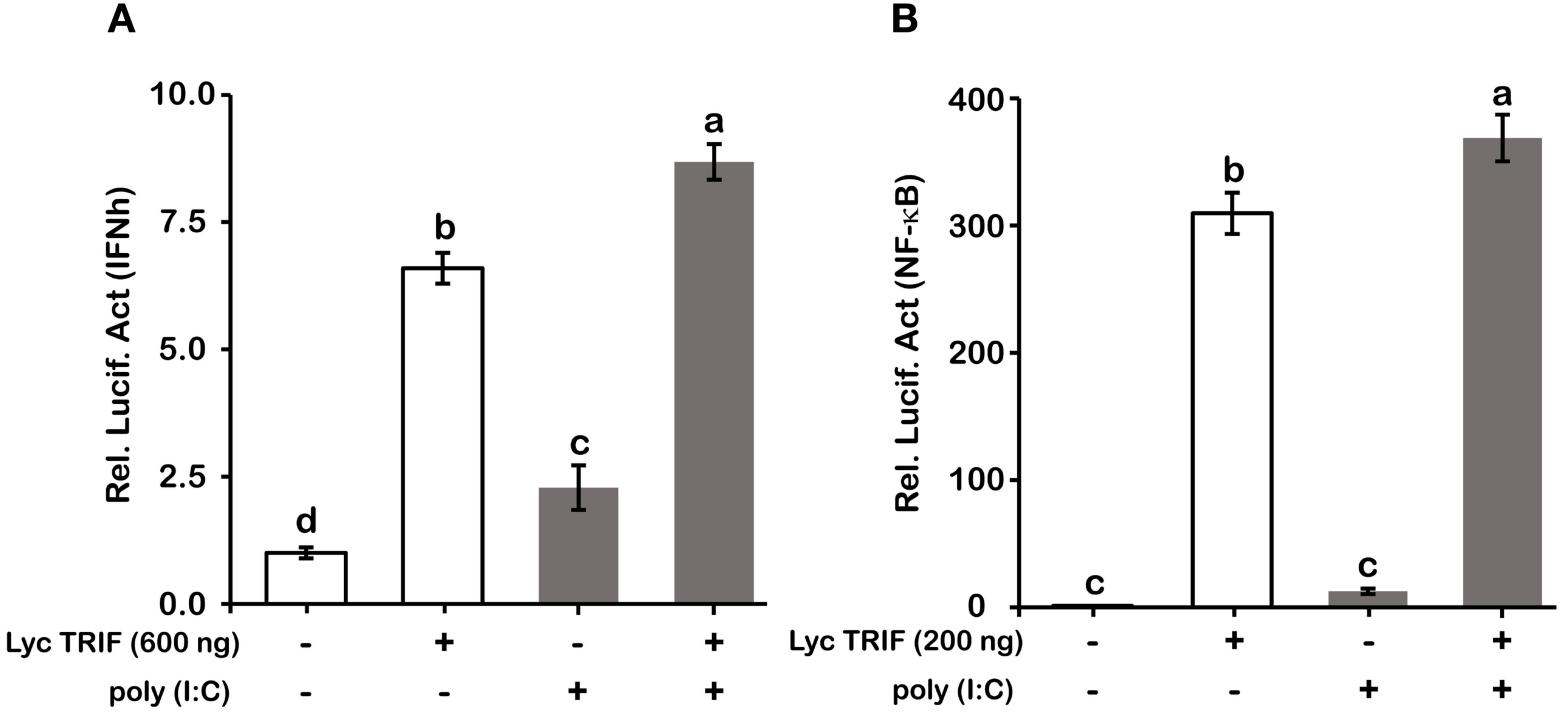
Poly (I:C) stimulation enhanced the promoter activities of IFNh **(A)** and NF-κB **(B)** upon LcTRIF overexpression. HEK293T cells cultured in 24-well plates overnight were cotransfected with TRIF expression vector together with the IFNh or NF-κB promoter luciferase reporter vector and 20 ng of pRL-TK. Twenty-four hours later, the cells were stimulated with poly (I:C). The luciferase activities were measured after 24 h post stimulation. Values (mean ± SD of three independent experiments with 4 technical replicates each) in bars that have the same superscript letter are not significantly different (*P* > 0.05, Tukey's test).

Type I IFN and NF-κB are commonly regarded as important inflammatory factors involved in virus-induced innate immune responses. To further elucidate the mechanisms of LcTRIF in inducing the inflammatory response, a luciferase reporter assay was carried out to test the promoter activities of IFNh and NF-κB in response to LcTRIF overexpression. The results showed that IFNh promoter activity was significantly enhanced in HEK293T cells transfected with 200 ng LcTRIF plasmid, and this enhancement increased with the increasing dose of LcTRIF plasmid, with promoter activity reaching a peak at the 600 ng dose (Figure 7A). However, LcTRIF overexpression induced the activity of the NF-κB response promoter in a different way, whereby the NF-κB promoter activity was inhibited after initial enhancement by 200 ng of LcTRIF plasmid (Figure 7B). Moreover, the enhancement of IFNh and NF-κB promoter activities by LcTRIF overexpression was more pronounced in the case of poly (I:C) stimulation (Figure 8). These results indicated that LcTRIF plays an important role in the immune responses triggered by dsRNA virus infections in macrophages of large yellow croaker.

### Activation of LcTRIF by Poly (I:C) Inhibited PA-Induced Lipid Accumulation in Hepatocytes

**Figure 9:**
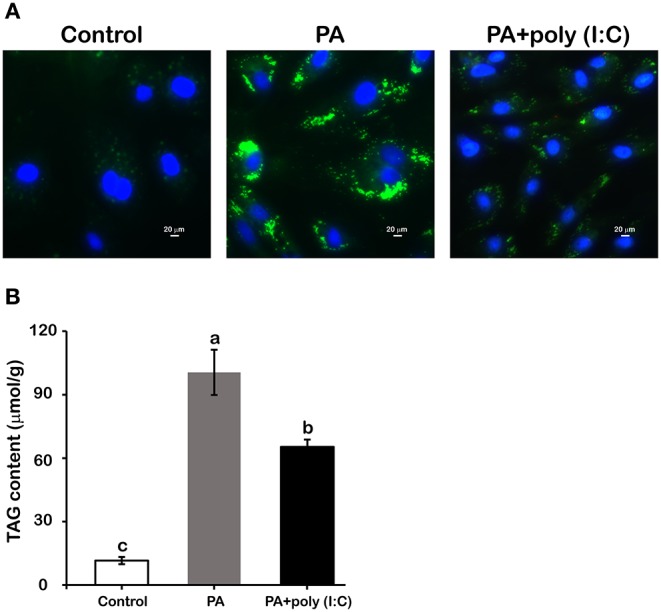
Activation of LcTRIF by poly (I:C) inhibited palmitic acid (PA)-induced hepatic lipid accumulation. Hepatocytes from large yellow croaker were incubated with or without PA (800 μM) and poly (I:C) (5 μg/mL) for 12 h. BODIPY 493/503 staining **(A)** and hepatic triglyceride content quantification **(B)** were performed. Values (mean ± SD of three independent experiments with 4 technical replicates each) in bars that have the same superscript letter are not significantly different (*P* > 0.05, Tukey's test).

The TRIF-dependent TLR pathway is generally believed to play a crucial role in regulating the inflammatory response in immune cells. Recently, increasing number of studies have suggested that the TRIF-dependent TLR pathway also plays a distinct role in lipid metabolism in metabolic cells (8–10). To investigate the specific role of LcTRIF in lipid metabolism, hepatocytes from large yellow croaker were incubated with or without PA and poly (I:C). BODIPY 493/503 staining showed that more lipid droplets accumulated in the hepatocytes incubated with PA than in the control group, whereas activation of the TRIF-mediated pathway by poly (I:C) inhibited the hepatic lipid accumulation induced by PA (Figure 9A). Quantification of extracted hepatic lipids showed that poly (I:C) stimulation significantly suppressed the PA-induced increase in TAG content (Figure 9B) (*P* < 0.05).

### LcTRIF Inhibited PA-Induced Hepatic Lipid Accumulation by Suppressing SCD1 Expression

**Figure 10:**
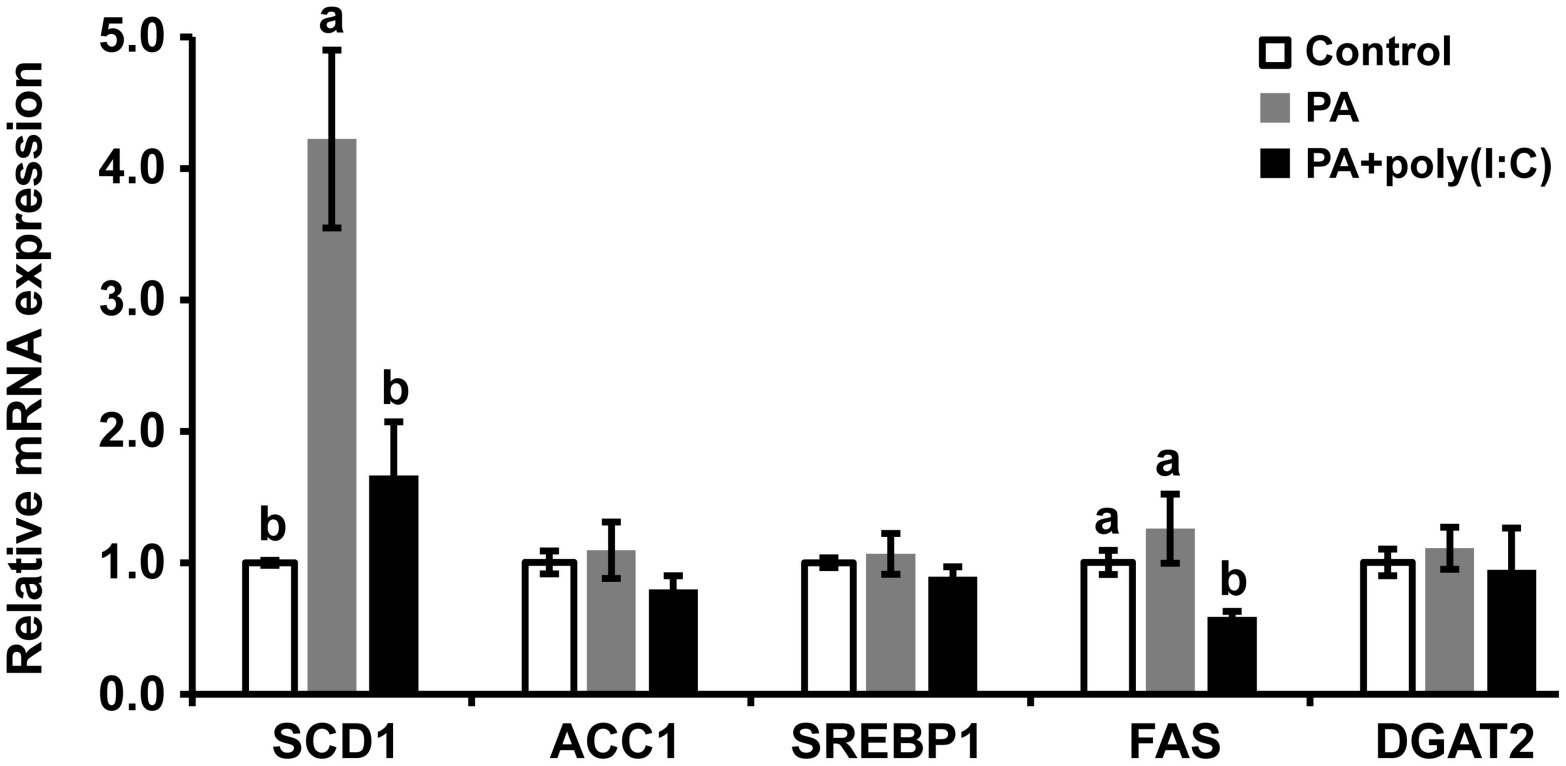
Activation of LcTRIF by poly (I:C) inhibited palmitic acid (PA)-induced hepatic SCD1 mRNA expression. Hepatocytes from large yellow croaker were incubated with or without PA (800 μM) and poly (I:C) (5 μg/mL) for 6 h, and the mRNA expression of hepatic SCD1, ACC1, SREBP-1, FAS, and DGAT2 was examined by qRT-PCR. Values (mean ± SD of three independent experiments with 4 technical replicates each) in bars that have the same superscript letter are not significantly different (*P* > 0.05, Tukey's test). ACC1, Acetyl-CoA carboxylase 1; DGAT2, Diacylgycerol acyltransferase 2; FAS, Fatty acid synthase; SCD1, Stearoyl–coenzyme A (CoA) desaturase 1, SREBP1, Sterol-regulatory element binding protein 1.

**Figure 11:**
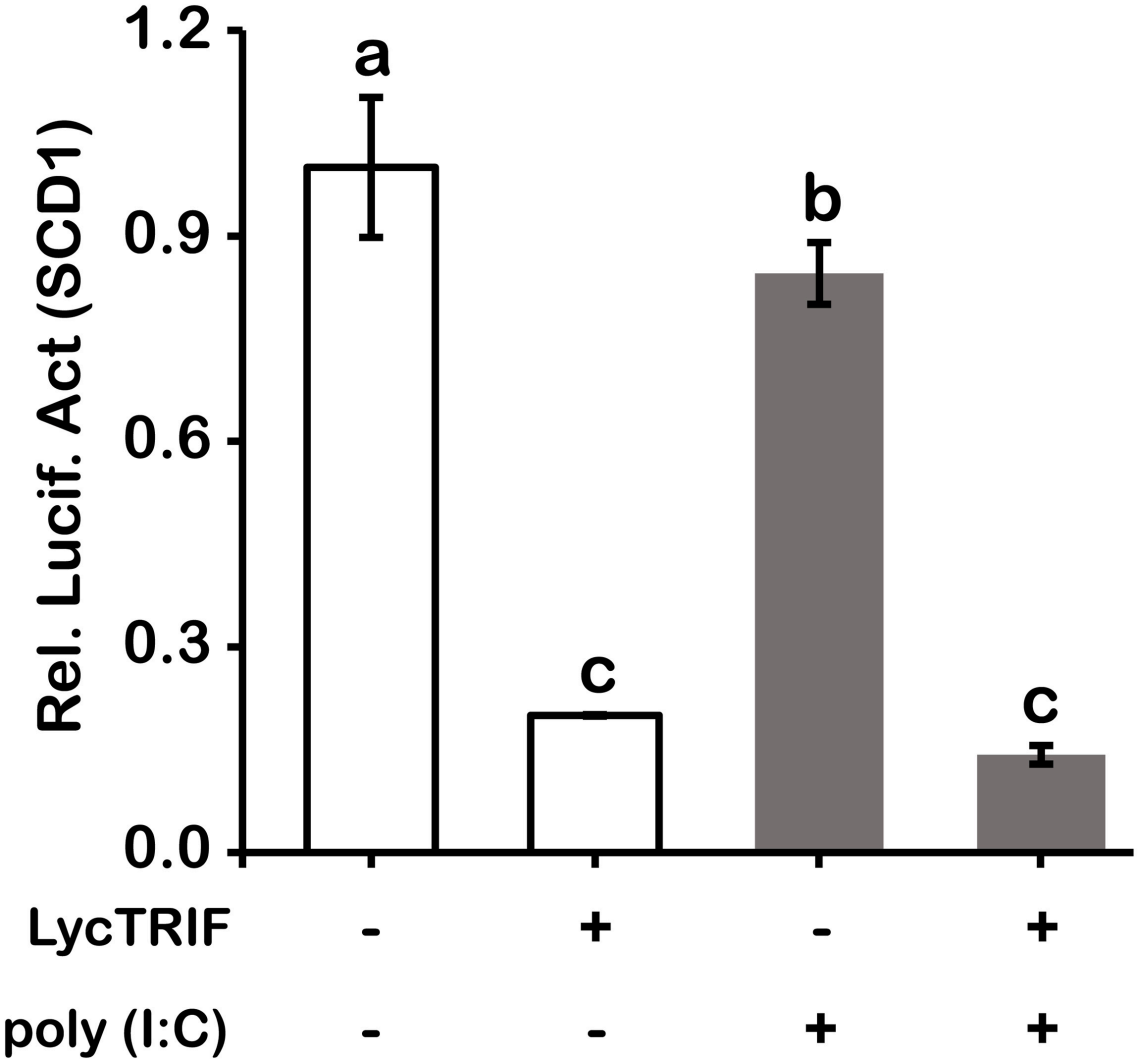
Overexpression of LcTRIF inhibited the promoter activity of SCD1. HEK293T cells cultured in 24-well plates overnight were cotransfected with the TRIF expression vector together with the SCD1 promoter luciferase reporter vector and 20 ng of pRL-TK. Twenty-four hours later, the cells were treated with or without poly (I:C) stimulation. The luciferase activities were measured after 24 h post stimulation. Values (mean ± SD of three independent experiments with 4 technical replicates each) in bars that have the same superscript letter are not significantly different (*P* > 0.05, Tukey's test).

To further investigate the mechanism of how LcTRIF affected hepatic lipid accumulation in large yellow croaker, the expression of genes involved in lipogenesis was detected. The results showed that PA incubation significantly increased *SCD1* expression in hepatocytes, and the activation of *LcTRIF* by poly (I:C) attenuated this induction (*P* < 0.05). However, PA and/or poly (I:C) treatment had no effect on the expression of other lipogenesis-related genes, such as *ACC1, SREBP1, FAS*, or *DGAT2* (*P* > 0.05) (Figure 10). Since LcTRIF inhibited SCD1 expression, we next examined whether this effect occurred at the promoter region of SCD1. The results from the luciferase report assay demonstrated that overexpression of LcTRIF in HEK293T cells significantly inhibited the promoter activity of SCD1, and this inhibition was more pronounced in poly (I:C) stimulation condition (Figure 11). These results indicated that the inhibition of hepatic lipid accumulation by LcTRIF might be partially attributed to the suppression of SCD1 expression.

### LcTRIF Was Distributed in the Cytoplasm

**Figure 12:**
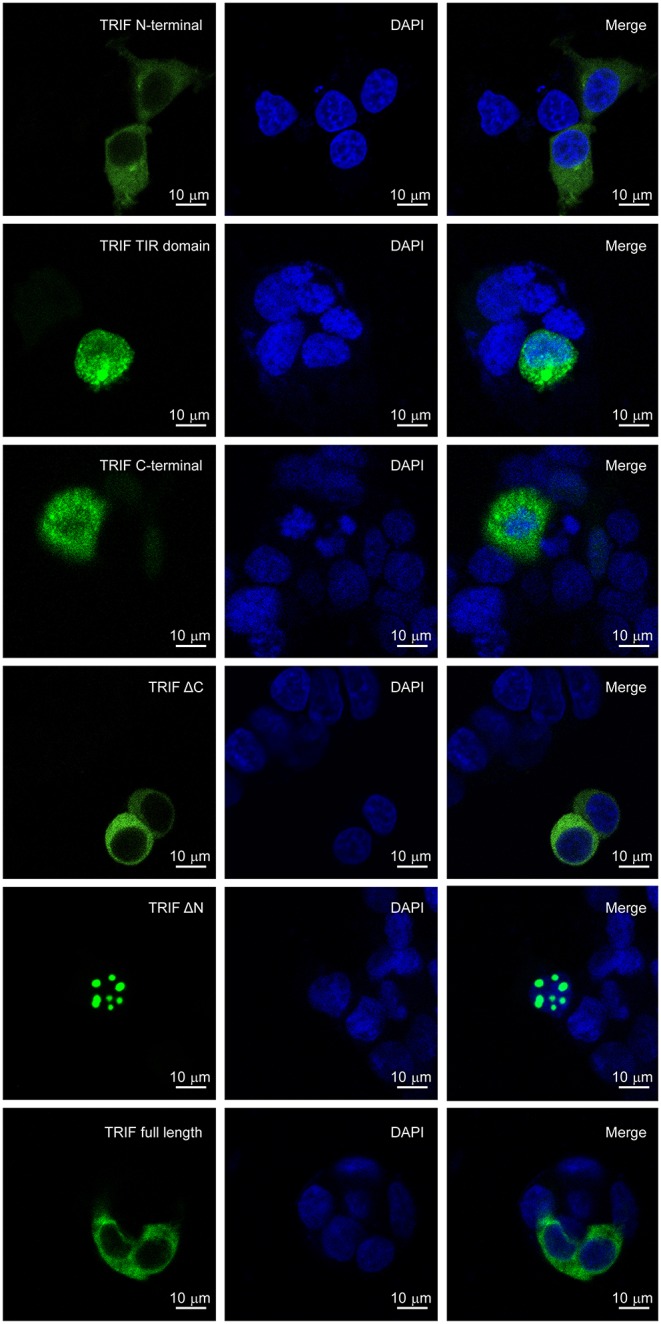
Localization of truncated forms of LcTRIF. Five truncated forms of pcDNA3.1-EGFP-TRIF fusion vectors including the TRIF N terminus (1–350 aa), TIR domain (351–471 aa), C terminus (472–601 aa), ΔC (1–471 aa), ΔN (351–601 aa), or TRIF (1–601 aa) were transfected into the HEK293T cells, respectively. After 24 h, the cells were stained with DAPI and then visualized by confocal laser microscopy.

The subcellular localization of LcTRIF was predicted by online software (http://www.csbio.sjtu.edu.cn/bioinf/euk-multi-2/), which indicated that LcTRIF was localized in the cytoplasm. To further investigate whether LcTRIF was localized in the cytoplasm, HEK293T cells were transfected with the pcDNA3.1-EGFP-TRIF fusion vector and then visualized by confocal microscopy. The results shown that LcTRIF was diffusely present in the cytoplasm. However, the truncated LcTRIF segments had different localizations. The truncated segments with only the C terminus or the TIR domain was distributed in the entire cell. The segment spanning the C terminus and the TIR domain (ΔN) was localized in the nucleus. The truncated segment with only the N terminus or the segment spanning the N terminus and the TIR domain (ΔC) was exclusively distributed in the cytoplasm, which showed the same subcellular localization as the full length protein (Figure 12). These results revealed that large yellow croaker TRIF was a cytoplasm-localized protein and that the N terminal sequence might contribute to its unique subcellular localization.

## Discussion

In the current study, the *TRIF* gene of large yellow croaker was cloned and characterized and was shown to share 35.30% identity with its counterpart in zebrafish. The deduced amino acid sequence of *LcTRIF* displayed a typical TIR domain that was highly conserved with the TIR domains of other fish species. All the teleost TRIF clustered together and separated from those of birds, reptiles, and mammals in a phylogenetic tree constructed based on the TIR domains. Compared with mammalian homologs, large yellow croaker TRIF contained proline in box 2 of the TIR domain, which is conserved in mammalian TRIF, TIRAP, and MyD88 and is essential for TRIF- and MyD88-mediated signal transduction (5, 32). However, the proline-rich domains close to the N terminus and C terminus in mammalian TRIF were not found in large yellow croaker TRIF. The lack of N-terminal and C-terminal proline-rich domains has also been reported for the zebrafish TRIF and orange spotted grouper TRIF (18, 19). Previous studies have demonstrated that TLRs and its adaptors had specific localization in the cells for their functions performed (18, 33, 34). To determine which sequence motif of LcTRIF is responsible for its localization, TRIF-EGFP fusion vectors and its truncated forms were constructed. The confocal laser microscopy analysis showed that large yellow croaker TRIF was uniformly distributed over the cytoplasm, and the N terminal sequences might contribute to its subcellular localization.

*In vivo*, upon poly (I:C) stimulation, the mRNA expression of *LcTRIF* was significantly upregulated in immune-related tissues at the early stage of injection. *In vitro*, the mRNA expression of *LcTRIF* was significantly but not dramatically upregulated in macrophages treated with poly (I:C). A similar expression pattern of *TRIF* in response to poly (I:C) stimulation has also been reported in kidney cells of grass carp (20). The activation of *LcTRIF* by poly (I:C) significantly elevated the mRNA levels of genes involved in inflammatory responses, and *LcTRIF* knockdown suppressed the poly (I:C) induced increase in the expression of these genes, which indicated that LcTRIF plays an important role in the immune responses triggered by dsRNA virus infections in macrophages of large yellow croaker. In addition, the results from the luciferase reporter assay showed that the promoter activities of IFNh and NF-κB were significantly increased in LcTRIF overexpression cells, and this increase was more pronounced in the case of poly (I:C) stimulation, which illustrated that LcTRIF triggers both the IFNh and NF-κB pathways in response to virus infections. These results agreed well with previous studies in mammals (5, 15) and fish (33, 34) demonstrating that TRIF-dependent pathways activate both type I IFN and NF-κB responses to protect the host from virus infections. Moreover, LcTRIF induced IFNh and NF-κB promoter activities in two different ways, which suggested that there was a negative feedback effect of the large yellow croaker TRIF-mediated NF-κB pathway.

Of particular interest was the distinct role of LcTRIF in hepatic lipogenesis of large yellow croaker. To date, TRIF homologs have been identified in several fish species, and their functions in antiviral immune responses have been investigated (16, 18–20). However, the potential role of TRIF in the lipid metabolism of fish has not been reported. In the present study, the activation of *LcTRIF* by poly (I:C) suppressed PA-induced hepatic lipid accumulation, and the inhibitory effects of LcTRIF on lipid accumulation might be due to the suppression of SCD1 expression. SCD1 is a key enzyme in lipogenesis, and catalyzes the formation of mono-unsaturated fatty acids (MUFA) from saturated fatty acids (SFA). MUFA are major substrates for TAG biosynthesis. Studies have showed that *SCD1*-deficient mice have significant lower hepatic TAG content than wild-type mice (35–38). The expression of SCD1 is generally regulated by several key metabolic transcription factors, including peroxisome proliferator-activated receptor α, sterol regulatory element-binding protein transcription factor 1c and liver X receptor (39, 40). Apart from these metabolic transcription factors, IRF3, a downstream transcription factor of TRIF, also plays a pivotal role in regulating SCD1 expression at the transcriptional level. Activation of TRIF by poly (I:C) induces phosphorylation of IRF3, which directly binds to the SCD1 promoter and blocks its transcription. The attenuation of SCD1 expression ameliorates hepatic lipid accumulation (10, 41). It has been speculated that the inhibitory effects of LcTRIF on SCD1 promoter activity in large yellow croaker is also mediated by IRF3. Further studies will be performed to verify this speculation. Moreover, the suppression of SCD1 expression by the LcTRIF-mediated pathway may also be considered an immune response, which limits viral assembly by counteracting virus-hijacked host lipogenesis (42, 43).

In summary, this study reported the identification and characterization of the *TRIF* gene from large yellow croaker, which had certain unique characteristics compared with its mammalian homologs. Activation of LcTRIF by poly (I:C) induced inflammatory response by promoting activation of the IFNh and NF-κB pathways in macrophages. In addition, LcTRIF activation inhibited PA-induced hepatic lipid accumulation by suppressing the SCD1 expression, suggesting a potential role of the TRIF-mediated signaling pathway in regulating lipogenesis in non-immune cells of fish. These results may contribute to the development of management strategies for defense against virus infections and alleviate abnormal lipid accumulation and inflammation induced by the use of HFD in teleosts.

## Data Availability Statement

All datasets generated for this study are included in the manuscript/Supplementary Files.

## Ethics Statement

The animal study was reviewed and approved by Institutional Animal Care and Use Committee of Ocean University of China.

## Author Contributions

KM, QA, and SZ designed the research. SZ and XXu conducted the research. SZ and XXi analyzed the data. SZ wrote the paper. SG provided language help. All authors reviewed and approved the final manuscript.

### Conflict of Interest

The authors declare that the research was conducted in the absence of any commercial or financial relationships that could be construed as a potential conflict of interest.
