# Supplementary material for: TIR Domain-Containing Adaptor-Inducing Interferon-β (TRIF) Participates in Antiviral Immune Responses and Hepatic Lipogenesis of Large Yellow Croaker (Larimichthys Crocea)
Source: Front Immunol. 2019 Oct 30;10:2506. doi: 10.3389/fimmu.2019.02506 (PMC6831525; doi:10.3389/fimmu.2019.02506)
Supplement: Supplementary file 1 [file Data_Sheet_1.docx]

Table 1 Primer pair sequences for cloning of TRIF from large yellow croaker

| Primer | Purpose | Forward (5'-3') | Reverse (5'-3') |
| --- | --- | --- | --- |
| TRIF-cDNA | Partial cDNA cloning | CAGGGGACGGGACTAAGAGA | GGTTTTGTTGCTGAGCCCTG |
| TRIF-RACE-1 | 3' and 5' RACE | CTCGTATGACTGTGGGGTTGGGTG | GCTCCTTCCAGGGATGGCAAAAT |
| TRIF-RACE-2 | 3' and 5' RACE | GACCAACTTTAGGCACTAGGGAGC | TGAAGCAGGCATCATGGGTAGAA |
| TRIF-RACE-3 | 3' and 5' RACE | TTTATTTCCCATCTCCCGTGACA | CAACTTAATGTTCTCTGCGGGGA |
| TRIF-FL | Full length cloning | AAGCAGTGGTATCAACGCAG | AAAATAAATGAATGAATTTATTGGT |

TRIF, TIR domain–containing adaptor-inducing interferon-β; RACE, rapid amplification of cDNA ends.

Table 2 Primer pair sequences for quantitative real-time PCR

| Primer | Forward (5'-3') | Reverse (5'-3') |
| --- | --- | --- |
| TRIF | TACAATACTGTTATCCCTCTGCTGC | TCTCTTCTGTTTTCTAATCCTCGCG |
| SCD1 | TGTTTCGTGGTAAGTGCTTTAGGAG | CGTCCTTTCTCAATAACATCGGG |
| FAS | AATCCTAACCCTGACATTCCTGCC | TACCTGCCCACTGTGTTCCCATAC |
| SREBP1 | TCTCCTTGCAGTCTGAGCCAAC | TCAGCCCTTGGATATGAGCCT |
| DGAT2 | TTCGGTGCTTTCTGCAACTTCG | AAGGATGGGGAAGCGGAAGT |
| ACC1 | GACTTGGCGGAATACCTACTGG | GCTTGCTGGATGATCTTTGCTT |
| IFNh | TGATTGGCTCAGACACTACGG | TGTTCGGTCTTGACGGTGTC |
| IRF3 | TTTTCAGAATCTGCTGCCCACC | GCAACGCCTGTCCTCCAATCAC |
| IL-1β | CATAGGGATGGGGACAACGA | AGGGGACGGACACAAGGGTA |
| TNFα | CGTCGTTCAGAGTCTCCTGC | TGTACCACCCGTGTCCCACT |
| β-actin | GACCTGACAGACTACCTCATG | AGTTGAAGGTGGTCTCGTGGA |

Abbreviations and GenBank Accession number: TRIF, TIR domain–containing adaptor-inducing interferon-β, MH820380.1; SCD1, stearoyl–coenzyme A (CoA) desaturase 1, KP202156.1; FAS, fatty acid synthase, KP889061.1; SREBP1, sterol-regulatory element binding proteins 1, XM_010730705.3; DGAT2, diacylgycerol acyltransferase 2, KJ563922.1; ACC1, acetyl-CoA carboxylase 1, XM_027273319.1; IFNh, interferon h, KU144879.1; IRF3, interferon regulatory factors 3, NM_001303387.1; IL-1β, interleukin 1β, KJ459927.1; TNFα, tumor necrosis factor α, EF070393.1.

Table 3 Primer pair sequences for plasmid construction

| Primer | Sequence (5'-3') |  |
| --- | --- | --- |
| TRIF-CDS-F | ATGAGCCACGAGGGAGAAGA |  |
| TRIF-CDS-R | CTATTGCTCATCTAAATCATCTTTG | |
| TRIF-pCS2-F | CGATTCGAATTCAAGGCCTCTCGAGATGAGCCACGAGGGAGAAGA | |
| TRIF-pCS2-R | CTCACTATAGTTCTAGAGGCTCGAGCTATTGCTCATCTAAATCATCTTTG | |
| TRIF-N-PCS2-F | CGATTCGAATTCAAGGCCTCTCGAGATGAGCCACGAGGGAGAAG | |
| TRIF-N-PCS2-R | CTCACTATAGTTCTAGAGGCTCGAGCTAAAATTGTGCCTCTTCCTTC | |
| TRIF-TIR-PCS2-F | CGATTCGAATTCAAGGCCTCTCGAGATGTACGCGTTTGTCATCTTTCAT | |
| TRIF-TIR-PCS2-R | CTCACTATAGTTCTAGAGGCTCGAGCTACCTGCTCTCATCGAGTG | |
| TRIF-C-PCS2-F | CGATTCGAATTCAAGGCCTCTCGAGATGAGCTTTGAGAAAAAAATAAAAGCATC | |
| TRIF-C-PCS2-R | CTCACTATAGTTCTAGAGGCTCGAGCTATTGCTCATCTAAATCATC | |
| NF-kB-pGL3-F | TCTTACGCGTGCTAGCCCGGGCAGAGTTGGGACACGGGCTG | |
| NF-kB-pGL3-R | GCCAAGCTTACTTAGATCGCAGATCGATGCACACACACACACACTCTCT | |
| IFNh-pGL3-F | TCTTACGCGTGCTAGCCCGGGCAGGCTTTTTGTTTTGTTCATTTCG | |
| IFNh-pGL3-R | GCCAAGCTTACTTAGATCGCAGATCGATGAAGATGCCGAAGATGACG | |
| SCD1-pGL3-F | TCTTACGCGTGCTAGCCCGGGCAACTCACCCTTTGCTTTCTCTGTC | |
| SCD1-pGL3-R | GCCAAGCTTACTTAGATCGCAGATCCGTGGCAGCTGGTTTAGTGAT | |
| TRIF- pcDNA3.1-EGFP-F | GGATCCACTAGTCCAGTGTGGTGGAATGAGCCACGAGGGAGAAGA | |
| TRIF- pcDNA3.1-EGFP-R | GCCACTGTGCTGGATATCTGCAGAATTGCTCATCTAAATCATCTTTG | |
| TRIF-N-pcDNA3.1-EGFP-F | GGATCCACTAGTCCAGTGTGGTGGAATGAGCCACGAGGGAGAAG | |
| TRIF-N- pcDNA3.1-EGFP-R | GCCACTGTGCTGGATATCTGCAGAAAAATTGTGCCTCTTCCTTC | |
| TRIF-TIR- pcDNA3.1-EGFP-F | GGATCCACTAGTCCAGTGTGGTGGAATGTACGCGTTTGTCATCTTTCAT | |
| TRIF-TIR- pcDNA3.1-EGFP-R | GCCACTGTGCTGGATATCTGCAGAACCTGCTCTCATCGAGTG | |
| TRIF-C- pcDNA3.1-EGFP-F | GGATCCACTAGTCCAGTGTGGTGGAATGAGCTTTGAGAAAAAAATAAAAGCATC | |
| TRIF-C- pcDNA3.1-EGFP-R | GCCACTGTGCTGGATATCTGCAGAATTGCTCATCTAAATCATC | |

TRIF, TIR domain–containing adaptor-inducing interferon-β; NF-κB, nuclear factor-kappa B; IFNh, interferon h; SCD1, stearoyl–coenzyme A (CoA) desaturase 1.

Table 4 siRNA sequences for RNA interference

| Primer | Forward (5'-3') | Reverse (5'-3') |
| --- | --- | --- |
| siTRIF-1 | GGGUGAGUCUCCUGAAGAUTT | AUCUUCAGGAGACUCACCCTT |
| siTRIF-2 | GCUCCUCAAUGCCCUCAUATT | UAUGAGGGCAUUGAGGAGCTT |
| siTRIF-3 | CCCUGUUAGUGAGUGUGAATT | UUCACACUCACUAACAGGGTT |
| siTRIF-4 | GCUCUUAUCAACUCCAUAATT | UUAUGGAGUUGAUAAGAGCTT |
| Negative control siRNA | UUCUCCGAACGUGUCACGUTT | ACGUGACACGUUCGGAGAATT |

siRNA, small interfering RNA; TRIF, TIR domain–containing adaptor-inducing interferon-β.


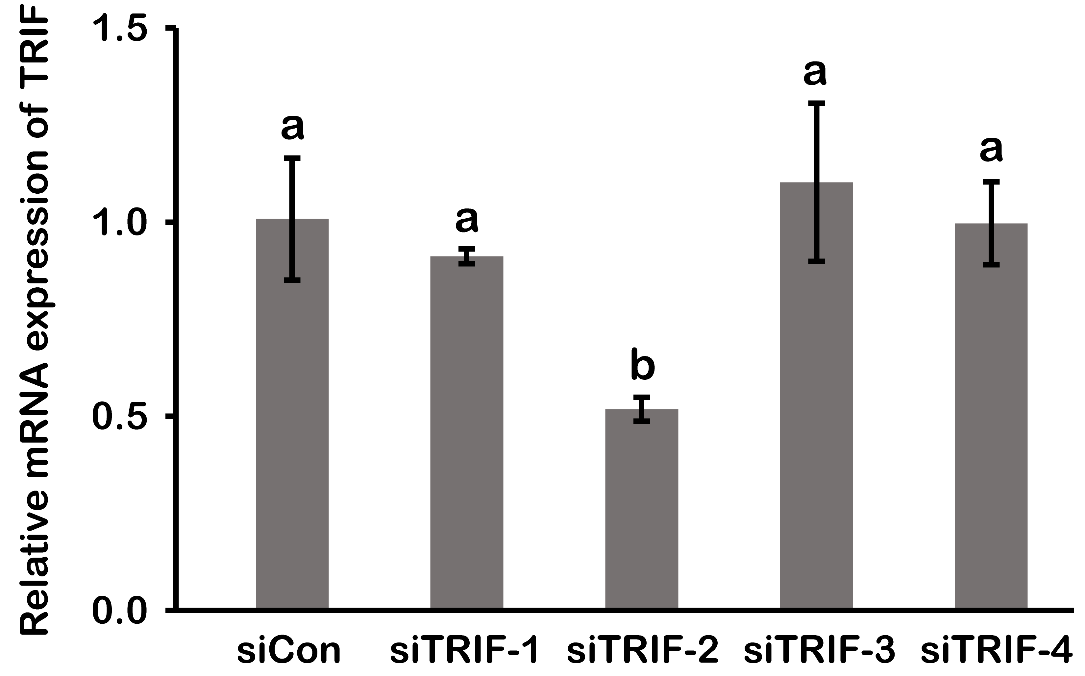


Figure 1. Knockdown of LycTRIF. Macrophages cultured in 6-well plates overnight were transfected with four TRIF siRNA candidates and negative control siRNA respectively. After 48 h, the knockdown efficiencies of siRNAs were examined by qRT-PCR. Values (mean ± SD of three independent experiments with 4 technical replicates each) in bars that have the same superscript letter are not significantly different (*P* > 0.05, Tukey’s test). siRNA, small interfering RNA; TRIF, TIR domain–containing adaptor-inducing interferon-β.
